# Microstructural but not macrostructural cortical degeneration occurs in Parkinson’s disease with mild cognitive impairment

**DOI:** 10.1038/s41531-022-00416-6

**Published:** 2022-11-09

**Authors:** Xueqin Bai, Tao Guo, Jingwen Chen, Xiaojun Guan, Cheng Zhou, Jingjing Wu, Xiaocao Liu, Haoting Wu, Jiaqi Wen, Luyan Gu, Ting Gao, Min Xuan, Peiyu Huang, Baorong Zhang, Xiaojun Xu, Minming Zhang

**Affiliations:** 1 Department of Radiology, The Second Affiliated Hospital, Zhejiang University School of Medicine, 310009 Hangzhou, China; 2 Department of Neurology, The Second Affiliated Hospital, Zhejiang University School of Medicine, 310009 Hangzhou, China

**Keywords:** Parkinson's disease, Dementia

## Abstract

This study aimed to investigate the cortical microstructural/macrostructural degenerative patterns in Parkinson’s disease (PD) patients with mild cognitive impairment (MCI). Overall, 38 PD patients with normal cognition (PD-NC), 38 PD-MCI, and 32 healthy controls (HC) were included. PD-MCI was diagnosed according to the MDS Task Force level II criteria. Cortical microstructural alterations were evaluated with Neurite Orientation Dispersion and Density Imaging. Cortical thickness analyses were derived from T1-weighted imaging using the FreeSurfer software. For cortical microstructural analyses, compared with HC, PD-NC showed lower orientation dispersion index (ODI) in bilateral cingulate and paracingulate gyri, supplementary motor area, right paracentral lobule, and precuneus (*P*_FWE_ < 0.05); while PD-MCI showed lower ODI in widespread regions covering bilateral frontal, parietal, occipital, and right temporal areas and lower neurite density index in left frontal area, left cingulate, and paracingulate gyri (*P*_FWE_ < 0.05). Furthermore, compared with PD-NC, PD-MCI showed reduced ODI in right frontal area and bilateral caudate nuclei (voxel *P* < 0.01 and cluster >100 voxels) and the ODI values were associated with the Montreal Cognitive Assessment scores (*r* = 0.440, *P* < 0.001) and the memory performance (*r* = 0.333, *P* = 0.004) in the PD patients. However, for cortical thickness analyses, there was no difference in the between-group comparisons. In conclusion, cortical microstructural alterations may precede macrostructural changes in PD-MCI. This study provides insightful evidence for the degenerative patterns in PD-MCI and contributes to our understanding of the latent biological basis of cortical neurite changes for early cognitive impairment in PD.

## Introduction

Cognitive impairment is one of the most common and debilitating non-motor symptoms of Parkinson’s disease (PD). Previous studies have reported that up to 19–42.5% of patients at initial diagnosis of PD have cognitive impairment^1–3^. The more recently recognized entity of mild cognitive impairment in PD patients (PD-MCI) represents individuals with early cognitive impairment for PD patients without impact on day-to-day functioning. Compared with PD patients with normal cognition (PD-NC), PD-MCI patients have a higher risk to develop dementia^4^, which can occur in up to 80% of PD patients over the long-term course of their disease^5^. Thus, detecting the early degenerative patterns of PD-MCI patients, who are at higher risk with dementia, is critical for understanding its pathophysiological mechanisms of cognitive impairment in PD.

Cognitive impairment in PD is thought to be related to cortical degeneration, which may result from the accumulation of Lewy-related pathology and Alzheimer’s disease (AD) like pathologies, e.g., amyloid-β and tau, in vulnerable cortical regions^6,7^. Previous studies have reported regional cortical degeneration in PD patients with cognitive impairment, which was represented as cortical atrophy measured by gray matter (GM) volume and cortical thickness using T1-weighted MRI^8–11^. However, these results are inconsistent with some other studies, which reported no significant morphological changes in PD-MCI patients^12–14^. The reason for this inconsistency may be that the morphological measurement with conventional structural MRI is poorly sensitive for early cognitive impairment in PD (e.g., PD-MCI) as it reflects GM atrophy caused by neuronal loss, a relatively late event in the process of cognitive impairment in PD patients.

A growing number of studies suggested that microstructural changes may precede the morphological changes in the development of neurodegeneration^15,16^. Recently, some studies reported that techniques sensitive to brain tissue microstructure are better suited to early detect brain changes in PD^17,18^. In contrast to T1-weighted structural MRI, diffusion-weighted MRI provides quantitative information about neural microstructure by measuring the diffusion properties of water molecules within tissues. An advanced multi-compartment diffusion model, Neurite Orientation Dispersion and Density Imaging (NODDI), can provide the information on the microstructure of the neurites by quantifying the neurite density index (NDI), orientation dispersion index (ODI) and the volume fraction of isotropic diffusion (*f*_iso_) within each voxel^19^. NDI describes the neurite density (higher values reflecting increased neurite density); ODI reflects the degree of neurite dispersion (lower values indicating decreased neurite orientation dispersion); and *f*_iso_ represents the proportion of cerebrospinal fluid (CSF) within a voxel (higher values suggesting increased isotropically diffusing freewater). The NODDI model, by separating out the isotropic CSF component of the diffusion signal, helps account for partial volume effects, which makes it a particularly well-suited tool for investigating cortical microstructural alterations. Considering the above-mentioned T1-weighted MRI reflecting the morphological changes at a relatively late state, we speculated that microstructural alterations reflected by NODDI might precede the macrostructural changes detected by T1-weighted MRI in PD-MCI, thus possibly provide information regarding pathophysiological processes in the cortex related to early cognitive impairment in PD.

This study aimed to investigate cortical micro/macrostructural degenerative patterns in PD-MCI by using diffusion-weighted imaging and T1-weighted imaging. Specifically, we applied a technique called gray matter based spatial statistics (GBSS), which takes advantage of the multi-compartment modeling of NODDI and allows for an unbiased GM specific voxel-wise statistical analysis of cortical microstructure.

## Results

The demographic and clinical characteristics of all subjects are presented in Table 1. According to the MDS Task Force 2012 level II criteria, 38 PD patients were classified as PD-MCI and 38 patients were classified as PD-NC. There was no significant difference in age, sex distribution, or years of education among the 3 groups (all *P* > 0.05; Table 1). UPDRS III score, H-Y stage, disease duration, LEDD, HAMD score, HAMA score, and RBDQ-HK score did not significantly differ between the PD-MCI group and PD-NC group (all *P* > 0.05; Table 1). Compared with the HC group and the PD-NC group, the PD-MCI group had poorer scores in all cognitive evaluations (all *P* < 0.05; Table 1). Besides, delayed recall of AVLT and memory cognitive domain scores in PD-NC group were significantly lower than those in HC group (both *P* < 0.05; Table 1).

**Table 1:** Demographic, clinical, and cognitive characteristics of the subjects.

	HC (*N* = 32)	PD-NC (*N* = 38)	PD-MCI (*N* = 38)	*P* value
Age (years)	59.11 ± 7.13	55.75 ± 9.23	59.69 ± 7.80	0.082^a^
Sex (M/F)	19/13	23/15	28/10	0.362^c^
Education (years)	10.91 ± 3.22	10.76 ± 4.01	9.50 ± 3.04	0.152^b^
UPDRS III score	–	18.58 ± 12.03	21.74 ± 12.84	0.272^b^
Disease duration (years)	–	4.24 ± 4.06	4.20 ± 2.75	0.952^b^
Hoehn and Yahr stage, median (range)	–	2 (1–3)	2 (1–3)	0.286^b^
Levodopa-equivalent dose (mg)	–	267.37 ± 242.80	299.66 ± 273.47	0.588^b^
HAMD	–	5.63 ± 4.87	6.16 ± 5.85	0.354^b^
HAMA	–	5.40 ± 4.27	6.02 ± 6.13	0.605^b^
RBDQ-HK	–	16.24 ± 13.13	21.32 ± 17.34	0.160^b^
MMSE	28.94 ± 1.11	28.95 ± 0.96	26.60 ± 2.62	<0.001^b,d,^***^f,^***
MoCA	27.22 ± 1.43	27.66 ± 1.36	20.84.6 ± 3.15	<0.001^b,d^***^f,^***
Executive function	0.04 ± 0.89	−0.02 ± 0.75	−0.51 ± 0.63	<0.001^b,d^**^,f^**
TMT-B	143.25 (45.51)	153.12 (63.63)	194.43 (75.46)	0.001^b,d^**^,f^**
Digit span backward	4.94 (1.37)	4.84 (1.59)	3.92 (1.24)	0.001^b,d^***^,f^**
Attention and working memory function	0.01 ± 0.82	0.08 ± 1.23	−0.95 ± 0.80	<0.001^a,d^***^,f^***
TMT-A	58.44 (23.34)	56.12 (19.27)	76.84 (35.14)	0.005^b,d^*^,f^**
SDMT	45.72 (9.95)	41.37 (11.99)	29.97 (10.72)	<0.001^a,d^***^,f^***
Visuospatial function	−0.06 ± 0.75	−0.03 ± 0.69	−0.48 ± 0.68	0.004^a,d^*^,f^**
CDT	8.44 (1.64)	8.53 (1.80)	7.44 (2.09)	0.023^b,d^**^,f^*
Cube copy	17.78 (3.75)	16.97 (3.83)	13.16 (6.00)	<0.001^b,d^***^,f^**
Language function	0.02 ± 0.91	−0.27 ± 0.70	−0.93 ± 0.91	<0.001^a,d^***^,f^**
BNT	26.75 (2.37)	25.58 (2.53)	22.76 (3.90)	<0.001^b,d^***^,f^***
AFT	19.44 (6.01)	17.61 (4.84)	15.00 (5.10)	<0.001^b,d^**^,f^*
Memory function	−0.04 ± 1.02	−0.33 ± 0.71	−1.63 ± 0.93	<0.001^a,d^***^,f^***
Delayed recall (AVLT)	7.31 (2.88)	5.90 (1.97)	4.37 (2.33)	<0.001^b,d^***^,e^*^,f^**
Delayed recall (MoCA)	3.78 (1.23)	3.57 (1.15)	1.76 (1.28)	<0.001^b,d^***^,f^***

*UPDRS* Unified Parkinson’s Disease Rating Scale part-III, *HAMD* Hamilton Depression Scale, *HAMA* Hamilton Anxiety Scale, *RBDQ-HK* Rapid Eye Movement Sleep Behavior Disorder Questionnaire - Hong Kong, *MMSE* Mini Mental State Examination, *MoCA* Montreal Cognitive Assessment, *TMT-B* Trail Making Test part B, *TMT-A* Trail Making Test part A, *SDMT* Symbol Digit Modality Test, *CDT* Clock-Drawing Test, *BNT* 30-item Boston Naming Test, *AFT* Animal Fluency Test, *AVLT* Auditory Verbal Learning Test.

*P < 0.05; **P < 0.01; ***P < 0.001.

^a^Comparison performed using a parametric test (Student’s *t* test or 1-way ANOVA, as appropriate).

^b^Comparison performed using nonparametric test (Mann–Whitney *U*-test or Kruskal–Wallis test, as appropriate).

^c^Comparison using chi-square test.

^d^The healthy control group versus the PD-MCI group.

^e^The healthy control group versus the PD-NC group.

^f^The PD-MCI group versus the PD-NC group.

### GBSS analyses

**Fig. 1:**
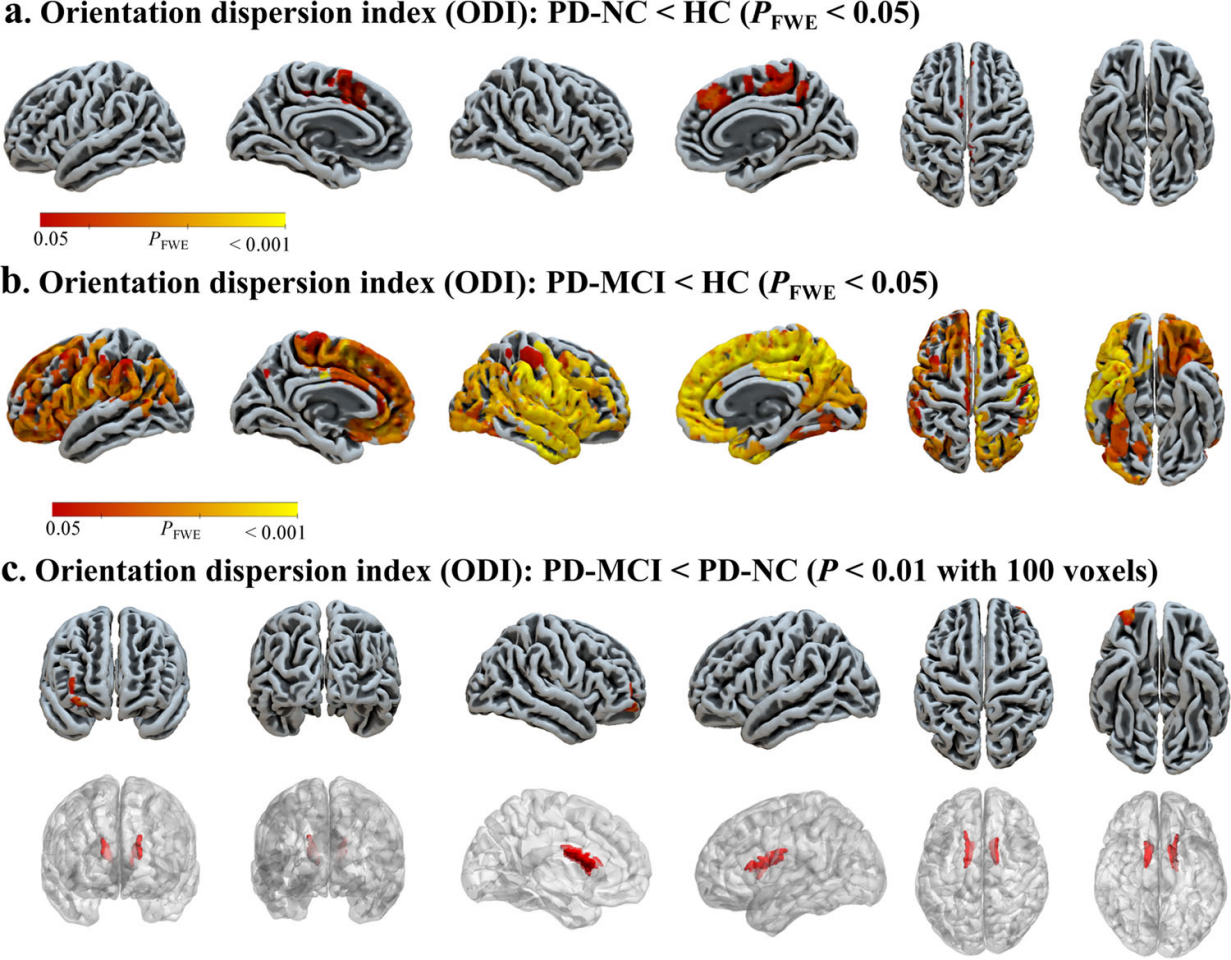
ODI comparisons among groups. Compared with HC, PD-NC patients showed lower ODI in bilateral cingulate and paracingulate gyri, supplementary motor area, right paracentral lobule and precuneus (**a**); PD-MCI patients showed widespread lower ODI throughout bilateral frontal, parietal, occipital and right temporal areas (**b**). Compared with PD-NC, PD-MCI patients showed reduced ODI in right frontal area and bilateral caudate nuclei for GBSS analyses with cluster-based thresholding method (voxel *P* < 0.01 and cluster >100 contiguous voxels) (**c**). HC healthy control, PD-NC Parkinson’s disease with normal cognition, ODI orientation dispersion index, PD-MCI Parkinson’s disease with mild cognitive impairment.

GBSS analyses were used to investigate cortical microstructural patterns of altered NODDI metrics in PD-NC and PD-MCI, adjusted for age, sex, and years of education. Compared with healthy controls (HC), PD-NC showed lower ODI in bilateral cingulate and paracingulate gyri, supplementary motor area, right paracentral lobule and precuneus (*P*_FWE_ < 0.05); while PD-MCI showed lower ODI in widespread regions covering bilateral frontal, parietal, occipital and right temporal areas (*P*_FWE_ < 0.05) (Fig. 1). Compared with PD-NC, PD-MCI showed reduced ODI in right frontal area and bilateral caudate nuclei for GBSS analyses with cluster-based thresholding method (voxel *P* < 0.01 and cluster >100 voxels) (Fig. 1), although no result survived when FWE method was employed.

**Fig. 2:**
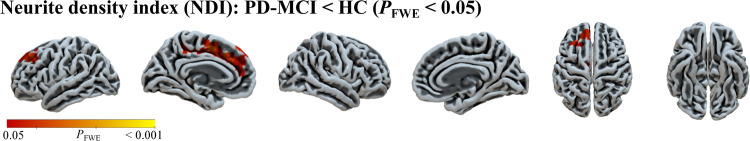
NDI comparisons among groups. Compared with HC, only PD-MCI patients showed reduced NDI predominantly in left frontal area, left cingulate and paracingulate gyri. HC healthy control, NDI neurite density index, PD-MCI Parkinson’s disease with mild cognitive impairment.

With respect to NDI, while there was no difference in the PD-NC group compared with HC, the PD-MCI group demonstrated lower NDI in left frontal area, left cingulate and paracingulate gyri (Fig. 2). There was no difference between PD-NC and PD-MCI.

For *f*_iso_, no significant difference in cortical microstructure was observed among groups.

Cortical microstructural alterations in PD-NC and PD-MCI, relative to HC, the patterns with cluster-based thresholding method were similar with those adjusted by using with FWE multiple-comparison correction (Supplementary Fig. 1).

### Cortical thickness analyses

No significant difference in whole-brain cortical thickness was observed among groups.

### Correlation analyses

**Fig. 3:**
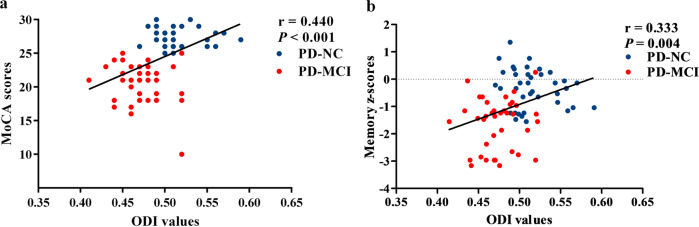
Correlation analyses in PD patients. The ODI values of the right frontal area were positively correlated with MoCA scores (**a**) and the memory performance (**b**) in all PD patients. ODI orientation dispersion index, MoCA Montreal Cognitive Assessment.

ODI values in the right frontal area, where significant difference was observed between PD-MCI and PD-NC, were significantly positively correlated with the MoCA scores (*r* = 0.440, *P* < 0.001) (Fig. 3a) and the memory performance of detailed neuropsychological tests (*r* = 0.333, *P* = 0.004) (Fig. 3b) in the whole PD patients, but not correlated with other specific cognitive domains (all *p* > 0.05). For MoCA subcategory domains, the ODI values were significantly positively correlated with the executive function, visuospatial ability, memory scores of the MoCA subcategory in the whole PD patients (*r* = 0.338, *P* = 0.003; *r* = 0.386, *P* = 0.001; *r* = 0.354, *P* = 0.002, respectively). There was no significant association between the ODI values and language, attention and working memory or orientational function of MoCA subcategory in the PD patients.

## Discussion

This study investigated the both micro/macro-structural degenerative patterns in PD patients with mild cognitive impairment. We had two main findings: (1) though PD-NC already showed microstructural degeneration in bilateral cingulate and paracingulate gyri, supplementary motor area, right paracentral lobule and precuneus, a more widespread degenerative pattern in PD-MCI was indicated, covering bilateral frontal, parietal, occipital and right temporal areas, and ODI values in the right frontal area were significantly positively correlated with the MoCA scores and the memory performance of detailed neuropsychological tests in the whole PD patients; (2) no macrostructural in cortical thickness was observed in PD-MCI and PD-NC. The finding of cortical microstructural alterations but not macrostructural changes in PD-MCI suggests cortical microstructural alterations may precede macrostructural changes in PD-MCI.

The ODI and NDI from NODDI model were closely correlated with the complexity of the dendritic arborization and the density/number of dendritic trees in the histologically validated studies^20,21^, which can reflect neurite orientation dispersion and neurite density respectively. Compared with HC, PD-NC showed reduced ODI in bilateral cingulate and paracingulate gyri, supplementary motor area, right paracentral lobule and precuneus, predominantly in the sensorimotor areas, suggesting cortical neurite degeneration in PD patients. Pathologically, PD is characterized by the relentless accumulation of alpha-synuclein inclusions (Lewy neurites), which is progressively accumulated in the subcortical and cortical regions^22^. The over-accumulated alpha-synuclein in the brain has been reported to impair the outgrowth and branching of dendrites in cortex^23^, which may reduce the variability of neurite orientations and reflect the decreased ODI values observed in this study. Furthermore, compared with NC, PD-MCI showed widespread abnormal ODI, covering bilateral frontal, parietal, occipital and right temporal areas, which indicated a more widespread cortical degeneration pattern in PD-MCI. Additionally, PD-MCI showed regional lower NDI relative to HC, predominantly in left frontal area, cingulate and paracingulate gyri, reflecting the loss of neurite structures in gray matter. A previous animal study reported overexpression of alpha-synuclein triggers decreased dendritic density in the neocortex, which leads to the reduction of NDI^24^. Neurites, the main structure of synaptic connections, comprise computational circuitry of the brain, which is tightly linked to functional efficiency, such as cognitive processes^25^. Since ODI and NDI allow the quantification of neurite morphology in terms of its orientation distribution and density, reflecting the nature of their computation and hence their function, their loss in our study reflected by decreased ODI and NDI values in PD-MCI may be a structural correlate for early cognitive impairment in PD. Besides, our study found that the PD-MCI patents present widespread changes in ODI but only regional changes in NDI. A previous study used PD mouse model to investigate the changes of cortical dendrites during the process of dopamine depletion in PD^26^. The study reported that both elimination and formation of cortical dendritic spine were enhanced by dopamine depletion in PD model, which can lead to the significant changes of dendritic arborization coherence and only a modest decrease in total dendritic spine numbers^26^. The significant changes of dendritic arborization coherence and a modest decrease in dendritic spine numbers might lead to widespread changes in ODI and regional changes in NDI in PD patients. It is worth noting that a number of studies have disclosed a significant relationship between AD pathologies and cognitive decline in PD^6,7^, and significant evidence has demonstrated that AD pathologies have close association with lower cortical ODI and NDI in AD patients and model mice^16,27^. Taken together, we speculated that our findings of the cortical microstructural changes in PD-MCI might be associated with AD pathologies. Further studies are warranted to reveal the specific pathophysiological mechanisms underlining the microstructural changes.

A neuropathological study has evidenced that more severe pathological proteins accumulate in cortex in PD patients with cognitive impairment^6^. Our findings are consistent with the neuropathological evidence and suggest that the ODI and NDI has the ability to potentially reflect the integrity of cortical neurites underlying PD pathology burden. A previous study has demonstrated decreased ODI and NDI values in subcortical nuclei indicating neurodegeneration in these regions^28^; however, rare evidence has been reported for the cortical microstructural alterations in PD detected by NODDI, and the relationship between cortical NODDI changes and cognitive impairment in PD is unknown. Kamagata et al.^15^ evaluated cortical changes between 30 PD patients and 28 HC by using NODDI with GBSS and reported the ODI and NDI values in sensorimotor and other cortex were negatively correlated with disease severity in PD patients. Our study further investigated the cognition decline-related cortical degeneration, and found that PD-MCI had significantly reduced ODI in right frontal area and bilateral caudate nuclei. The function of frontal lobe and caudate nucleus in cognition has been well established^29,30^, specifically degenerative alterations (e.g., atrophy and functional connectivity alterations) in these regions were commonly found in PD patients with cognitive impairment^31,32^. As for the relationships between NODDI changes and cognitive performance, we found the ODI values within right frontal area were positive correlated with MoCA scores and the memory performance of detailed neuropsychological tests in the whole PD patients, but not correlated with other specific cognitive domain. The frontal cortex is thought to be important for memory function^33^ and previous studies reported that memory impairment was frequently affected in PD-MCI^2,34^. Consistent with our findings, several studies have reported the degeneration of the frontal area in PD patients with cognitive impairment, which were correlation with memory performance^11,35^. Our finding from the perspective of microstructure supported the assumption that the degeneration in the frontal area were correlated with memory dysfunction in PD patients. The presentations of cognitive deficits in PD-MCI are complex and heterogeneous, with a range of cognitive domains affected^36^. Different PD-MCI patients may suffer from different cognitive domain impairment, which may result in the relatively low statistical effects for specific cognitive measure and lead to the lack of association between the ODI values and the specific cognitive domain scores. However, the MoCA test comprises multiple cognitive domains, which reflects the composite cognitive deficit degree^37^ and may be more relevant to the comprehensive cortical degeneration reflected by the ODI values. Besides, for MoCA subcategory analyses, the ODI values were significantly positively correlated with the executive function, visuospatial ability, memory scores of the MoCA subcategory in PD patients. The correlations between ODI value and cognitive performance show some different when applying the MoCA subcategory or detailed neuropsychological tests to refer cognitive performance, we supposed that the different sensitivity and specificity of MoCA subtests and detailed neuropsychological assessments may contribute to this difference. Further studies would be necessary to identify the cognitive domain-specific cortical microstructural degenerative patterns.

In contrast to microstructural changes, no cortical thickness difference was detected with traditional macrostructural analyses suggesting that morphological changes may not an early biomarker to detect cortical degeneration in cognitive impairment in PD. Although previous studies of cortical morphological changes have reported considerable atrophy in PD patients with dementia, macrostructural studies in PD-MCI have largely inconsistent results. Some studies have identified various regional cortical atrophy, involving frontal, parietal, temporal, occipital, and limbic areas^10,11,38^, while other studies reported no significant morphological changes in PD-MCI^12,14^. We speculated that these inconsistencies might result from following reasons: (1) cortical thickness measures were considered as indirect indications of neuronal loss, a relatively late event in the progressive cortical degeneration, which makes it poorly sensitive for PD-MCI patients; (2) the difference of statistical effects that caused by various sample size and disease heterogeneity; (3) various definitions of PD-MCI and lacking of consistency in specific neuropsychological tests. Taken together, our findings of decreased ODI and NDI in the cortex with the absence of changes in cortical thickness indicate that microstructural degeneration of cortical neurites may precede macroscopically detectable neuronal loss in PD-MCI, which is consistent with histopathological studies suggesting that accumulation of pathology are thought to related to dystrophic neurites and synaptic dysfunction first, and eventually lead to neuronal loss during the degenerative process in PD^39,40^. In addition, these findings also demonstrated that the potential of NODDI as a microstructural measurement with NODDI may be more sensitive than macrostructural evaluation in the detection of cortical degeneration in PD-MCI.

There are several limitations in our study. Firstly, the relatively poor voxel resolution of the diffusion MRI limited the exploration of precise cortical microstructural characterization. However, estimation of the WM and CSF fraction and the skeletonization step in GBSS are designed to restrict statistical analysis to the center of cortical structure where risk of partial volume effect is relatively controlled. Secondly, the lack of the correction of the distortion coming from EPI’s oscillating gradients would have potential influence on diffusion metric estimation. Future studies acquiring opposite phase b0 images combining with the newer methodology (such as “top-up” algorithm in the FSL) would improve the correction of geometrical distortion. Furthermore, the cluster-based thresholding correction method with voxel *P* < 0.01 and 100 contiguous voxels is relatively liberal. Despite the relatively weak comparison, we also added the corresponding results for preliminary explorative analysis in the study. Finally, our study is a cross-sectional study with relatively small sample size. Further longitudinal studies with a larger sample size are required to clarify the utility of NODDI metrics in predicting or tracking the cognitive decline in PD.

In conclusion, widespread microstructural degeneration detected by NODDI-GBSS have close relationship with cognitive impairment in PD, which may precede macrostructural changes. NODDI-GBSS has been demonstrated as a sensitive and available approach to describe degenerative process of PD, and more relevant attempts are warranted. Our study provides insightful evidence for the degenerative patterns in PD-MCI and contributes to our understanding of the latent biological basis of cortical neurite changes for early cognitive impairment in PD.

## Methods

### Participants

This study was approved by the medical ethics committee of the Second Affiliated Hospital of Zhejiang University School of Medicine, and written informed consent was obtained from each participant. A total of 108 subjects, including 76 PD patients and 32 age, sex, education matched healthy controls (HC), were included in this study. Subjects with a history of other neurologic or psychiatric disorders, brain trauma, general exclusion criteria for MR scanning, or PD patients with dementia were excluded from this study. Parkinson’s disease with dementia (PDD) were diagnosed according to the clinical diagnostic criteria for PDD from the Movement Disorder Society Task Force^41^. The core features of PDD were cognitive impairment in more than one cognitive domain and deficits severe enough to impair daily life(the score of Activities and Daily Living scale^42^ ≥26 or the score of Functional Activity Questionnaire scale^43^ ≥6) for PD patients. All the PD patients were diagnosed according to the criteria of UK Parkinson Disease Society Brain Bank by a senior neurologist^44^. For PD patients who were under anti-Parkinsonian medication, the MR scanning and clinical assessments were performed on “drug-off status” (after withdrawing all anti-parkinsonian drugs at least 12 h) to minimize the potential pharmacological influences. Basic demographics and clinical information, including age, sex, years of education, Unified Parkinson’s Disease Rating Scale part-III (UPDRS-III) score, Hoehn–Yahr (H-Y) stage, disease duration, and levodopa equivalent daily dose (LEDD) were obtained from all PD patients. In addition, all PD patients were assessed for depressive symptoms using the Hamilton Depression Scale (HAMD), for anxiety trait using the Hamilton Anxiety Scale (HAMA), and for rapid eye movement sleep behavior disorder using the Rapid Eye Movement Sleep Behavior Disorder Questionnaire - Hong Kong (RBDQ-HK)^45^. For HC, basic demographic information, including age, sex, years of education, were recorded.

All the subjects underwent a comprehensive cognitive assessment including global cognitive function tests and a neuropsychological battery covering all five cognitive domains. Global cognitive function was assessed using Mini Mental State Examination (MMSE) and Montreal Cognitive Assessment (MoCA). The details of neuropsychological battery covering five domains are as follows: (1) executive function was assessed using Trail Making Test part B (TMT-B) and Digit span backward; (2) attention and working memory was evaluated using Trail Making Test part A (TMT-A) and Symbol Digit Modality Test (SDMT); (3) visuospatial function was assessed with the Clock-Drawing Test (CDT) and cube copying test from MoCA test; (4) language was measured with 30-item Boston Naming Test (BNT) and Animal Fluency Test (AFT); (5) Memory was evaluated using Auditory Verbal Learning Test (AVLT) with delayed recall and delayed recall subset of the MoCA test^46^. According to the level II diagnostic criteria recommended by the Movement Disorder Society (MDS) Task Force 2012, PD-MCI was diagnosed when impairment on at least two tests for detailed cognitive battery with scores 1.5 standard deviations (SDs) below the normative mean^36^, either within a single cognitive domain or across different cognitive domains. PD patients without dementia who did not meet the criteria for PD-MCI were classified as PD-NC.

### MRI acquisition

All subjects were scanned on a 3.0 Tesla MR imaging system (Discovery MR750, GE Healthcare) equipped with eight-channel head coil. Two-shell diffusion images were acquired using spin-echo echo-planar imaging sequence with 30 gradient directions for each non-zero *b* value (*b* value = 1000 s/mm^2^, 30 directions; *b* value = 2000 s/mm^2^, 30 directions); The sequence parameters were as follows: repetition time (TR) = 5000 ms; echo time (TE) = 94 ms; flip angle = 90°; field of view (FOV) = 256 × 256 mm^2^; matrix = 128 × 128; slice thickness = 4 mm; slice gap = 0 mm; 34 interleaved axial slices and acquisition time was 5 min 20 s. Three-dimensional T1-weighted images were acquired using fast spoiled gradient recalled sequence: TR = 7.3 ms; TE = 3.0 ms; inversion time (TI) = 450 ms; flip angle = 11; FOV = 260 × 260 mm^2^; matrix = 256 × 256; slice thickness = 1.2 mm; slice gap = 0 mm; 196 continuous sagittal slices and acquisition time was 5 min 53 s.

### Diffusion MRI preprocessing and GBSS analysis

Diffusion images were preprocessed using FMRIB Software Library (FSL, https://fsl.fmrib.ox.ac.uk/fsl/fslwiki/), including the brain extraction and the correction of eddy current distortion and inter-volume head motion^47^. After the data preprocessing, the resulting diffusion data were fitted to the NODDI model using the NODDI MatLab Toolbox (http://www.nitrc.org/projects/noddi_toolbox) to generate NDI maps, ODI maps, and *f*_iso_ maps^48^. The tensor metric fractional anisotropy (FA) was estimated with *b* = 1000 images by fitting using FSL’s “DTIFIT” tool. Then, All the NODDI parameter maps and FA maps were resampled to 2 × 2 × 2 mm to improve the resolution.

**Fig. 4:**
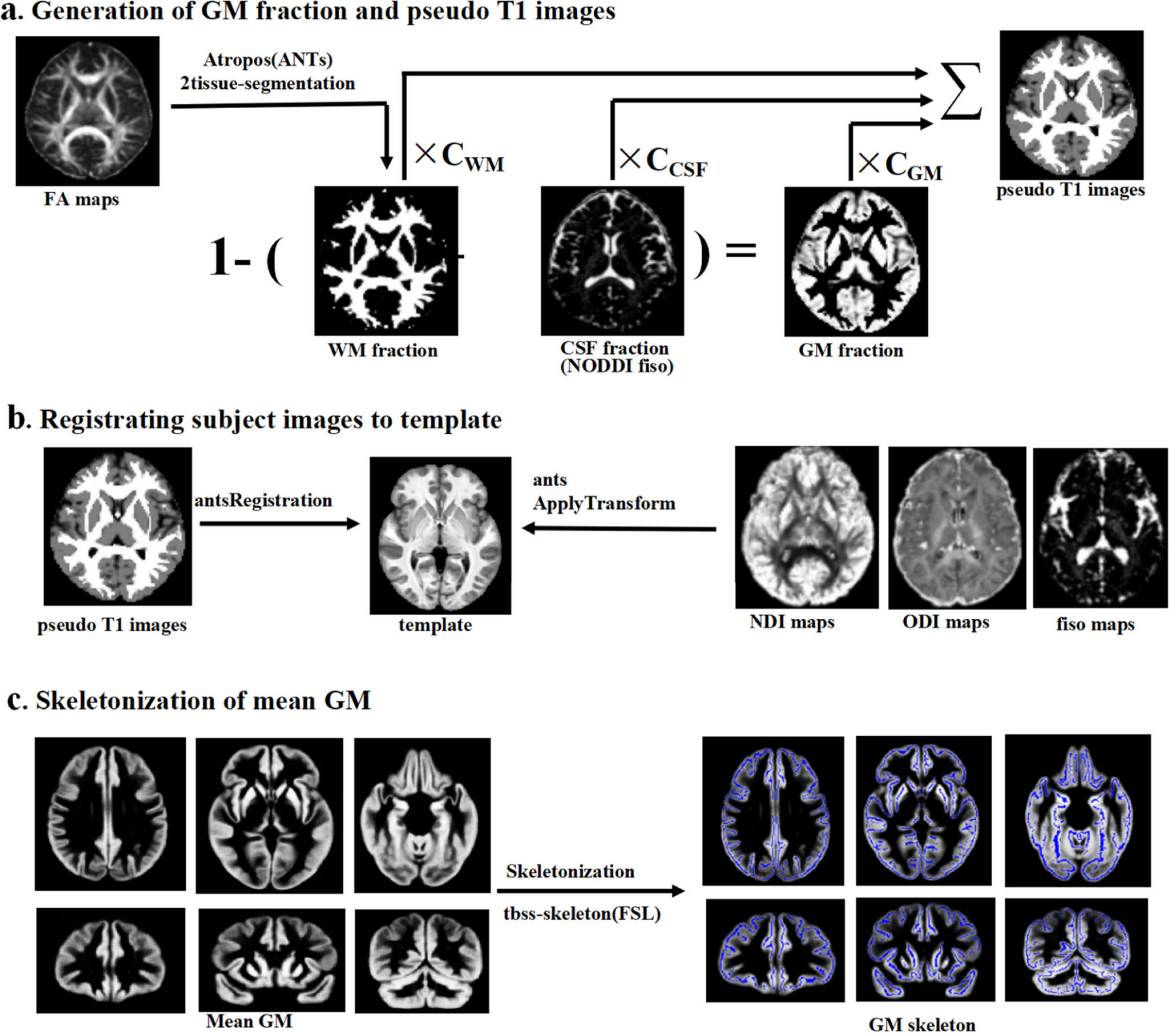
Processing steps of GBSS. **a** GM fraction maps were generated by subtracting the WM fraction (estimated by two-tissue class segmentation of resampled FA maps using Atropos segmentation tool) and the CSF fraction (resampled *f*_iso_ maps from NODDI) from one. The GM, WM, and CSF fraction were used to generate pseudo T1-weighted images. **b** Pseudo T1-weighted images from all subjects were registered to the OASIS-30_Atropos_template and all resampled NODDI maps and GM fraction maps were aligned to the template. **c** GM fraction images from all subjects were averaged to generate mean GM images, which were skeletonized using FSL’s tbss_skeleton tool. All NODDI parameters maps and GM fraction maps were projected onto this GM skeleton. GBSS gray matter-based spatial statistics, GM gray matter, WM white matter, FA fractional anisotropy, CSF cerebrospinal fluid, NODDI Neurite Orientation Dispersion and Density Imaging, NDI neurite density index, ODI orientation dispersion index, *f*_iso_ volume fraction of isotropic diffusion, FSL FMRIB Software Library.

GBSS is a statistical technique, adapts the tract-based spatial statistics (TBSS) framework, to allow voxel-wise analysis on GM^49^. A schematic of the processing steps is shown in Fig. 4. Firstly, we got CSF fraction from the resampled *f*_iso_ maps from NODDI and WM fraction maps, which were estimated by two-tissue class segmentation of resampled FA maps using Atropos segmentation tool in Advanced Normalization Tools (ANTs)^50^. Then, GM fraction maps were generated in the native diffusion space by subtracting CSF fraction and WM fraction from one. Each tissue segmentation maps were then multiplied by their respective tissue weighting (CSF = 0, GM = 1, WM = 2) and summed to generate “pseudo T1-weighted” images^49^. Next, each participant “pseudo T1-weighted” images were registered to OASIS-30_Atropos_template and the transformation matrix was generated. Native diffusion space NODDI parameter maps (NDI, ODI, and *f*_iso_) and GM fraction maps were aligned to the template space by applying the corresponding transformation. The GM fraction maps in template space from all participants were merged and then averaged to generate mean GM images, which were skeletonized using the tbss_skeleton tool in FSL. The GM skeleton was thresholded to only include voxels with GM fraction > 0.65 in > 70% of participants, which represented the centers of all GM common to the groups^51^. Finally, all NODDI parameters maps and GM fraction maps were projected onto this GM skeleton.

### Cortical thickness analysis

Cortical reconstruction and estimation of cortical thickness were performed using the FreeSurfer software (version 6.0.0, http://surfer.nmr.mgh.harvard.edu/fswiki); Briefly, the processing procedure included motion correction, removal of non-brain tissue, automated Talairach registration, segmentation of the subcortical white matter (WM) and deep gray matter volumetric structures, tessellation of the GM/WM boundary, intensity normalization, automated topology correction, and surface deformation^52^. Cortical thickness was estimated as the shortest distance between the GM / WM boundary and the pial surface at each vertex. Finally, cortical maps were generated following registration of all subjects’ cortical reconstructions to a common average surface and then smoothed using a 10-mm full width at half maximum kernel.

### Statistical analysis

Demographic and clinical data were analyzed using one-way analysis of variance (ANOVA) followed by post hoc Bonferroni correction for multiple comparisons, or Kruskal-Wallis test followed by Bonferroni method for multiple-comparison correction among three groups. Difference in sex distribution was compared using chi-square test. Between PD groups, variables were compared using the Student’s *t* test or Mann–Whitney *U*-test. Statistical significance was set to *P* < 0.05.

For both GBSS and cortical thickness analyses, inter-group comparisons were conducted (PD-NC versus NC; PD-MCI versus NC; and PD-MCI group versus PD-NC group) with age, sex, and years of education as covariates of no interests. GBSS voxel-wise analyses on the skeletonized NODDI parameter maps were performed with FSL’s randomize by using nonparametric permutation analyses (n = 5000 permutations). Threshold-free cluster enhancement was used for each permutation analysis to determine statistical significance fully corrected for multiple comparisons with family-wise error (FWE) method. *P*_FWE_ < 0.05 was considered as statistically significant for GBSS analyses. It is worth noting that, though significant different cortical degeneration patterns were observed when comparing each PD group with normal controls (Fig. 1), no result survived between PD groups when conservative FWE method was used to control type I error with increasing type II error, leading to the loss of subtle changes^53^. Therefore, in order to balance type I and type II errors^53^, an explorative cluster-based thresholding method with voxel *P* < 0.01 and 100 contiguous voxels was applied^54^.

Cortical thickness analyses were assessed vertex-wise using a GLM and the level of statistical significance was evaluated using a cluster-wise *P* (CWP) value correction procedure for multiple comparisons with cluster-based Monte–Carlo simulation using 5000 permutations. Clusters with CWP value < 0.05 were considered statistically significant for cortical thickness analyses.

Correlation analyses between the cortical NODDI value and cognitive performance (global cognition and each cognitive domain behavior) were performed in all PD patients, adjusting for age, sex, and years of education. The specific cognitive domain behavior was obtained in a two-step process^55^. First, the raw scores of the neuropsychological battery covering five domains were transformed to z scores. Second, the composite score of each cognitive domain was calculated by averaging the z scores of the corresponding two neuropsychological tests belonging to each specific cognitive domain^3^. To confirm the regional correlations, NODDI metric values within the areas showing statistically significant between PD-MCI and PD-NC from GBSS were extracted. Then, partial correlation analyses were performed between mean regional NODDI values of the identified areas and the global cognitive scores or each cognitive domain composite score. Besides, we further conducted the correlation analyses between the NODDI values and the MoCA subcategory score to explore the relationship between the cortical microstructure and different cognitive tests. For MoCA test, sets of questions were grouped together and labeled as evaluating executive function, visuospatial ability, memory, language, attention and orientation function^37^. Partial correlation analyses were performed between mean regional NODDI values of the identified areas and the MoCA subcategory scores.

### Reporting summary

Further information on research design is available in the Nature Research Reporting Summary linked to this article.

## Supplementary information

Supplementary materials

Reporting Summary

## Data Availability

The data used in this manuscript are available from the corresponding author upon reasonable request (e.g., reproducibility of research).

## Code availability

All code used in this study will be shared upon reasonable request to the corresponding author.
