# Supplementary material for: Microstructural but not macrostructural cortical degeneration occurs in Parkinson’s disease with mild cognitive impairment
Source: NPJ Parkinsons Dis. 2022 Nov 9;8:151. doi: 10.1038/s41531-022-00416-6 (PMC9646695; doi:10.1038/s41531-022-00416-6)
Supplement: Supplementary file 1 — Supplementary materials [file 41531_2022_416_MOESM1_ESM.pdf]

Supplementary material

Microstructural but not macrostructural cortical degeneration occurs in Parkinson's disease with mild cognitive impairment

Results

GBSS analyses with cluster-based thresholding method (voxel $P < 0.01$ and cluster > 100 voxels)

GBSS analyses were used to investigate cortical microstructural patterns of altered NODDI metrics in PD-NC and PD-MCI, adjusted for age, gender, and years of education. Compared with HC, PD-NC showed lower ODI in bilateral frontal areas, cingulate and paracingulate gyri, bilateral supplementary motor area and paracentral lobule, left temporal areas and right Fusiform gyrus (Supplementary Figure 1A); while PD-MCI showed widespread lower ODI throughout bilateral frontal, parietal, occipital and temporal areas (Supplementary Figure 1 B). Compared with PD-NC, PD-MCI showed reduced ODI in right frontal area and bilateral caudate nuclei for GBSS analyses with cluster-based thresholding method.

With respect to NDI, while there was no differences in the PD-NC group compared with HC, the PD-MCI group demonstrated regional lower NDI, predominantly in left frontal area, left cingulate and paracingulate gyri, supplementary motor area, precuneus, and middle occipital gyru (Supplementary Figure 1 C). There was no difference between PD-NC and PD-MCI.

For fiso, no significant difference in cortical microstructure was observed among groups.

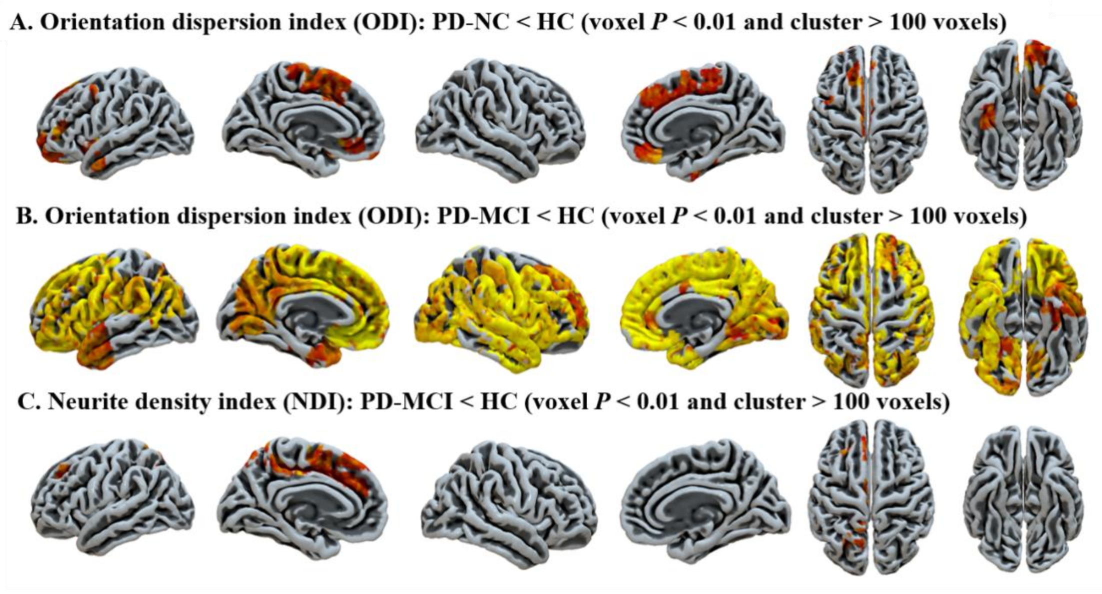

Supplementary Figure 1. Cortical microstructural analyses with cluster-based thresholding method. Compared with HC, PD-NC showed lower ODI in bilateral frontal areas, cingulate and paracingulate gyri, bilateral supplementary motor area and paracentral lobule, left temporal areas and right Fusiform gyrus (A); while PD-MCI showed widespread lower ODI throughout bilateral frontal, parietal, occipital and temporal areas (B). With respect to NDI, compared with HC, the PD-MCI group demonstrated regional lower NDI, predominantly in left frontal area, left cingulate and paracingulate gyri, supplementary motor area, precuneus, and middle occipital gyru (C).
