# Supplementary material for: Microstructural but not macrostructural cortical degeneration occurs in Parkinson’s disease with mild cognitive impairment
Source: NPJ Parkinsons Dis. 2022 Nov 9;8:151. doi: 10.1038/s41531-022-00416-6 (PMC9646695; doi:10.1038/s41531-022-00416-6)
Supplement: Supplementary file 2 — Reporting Summary [file 41531_2022_416_MOESM2_ESM.pdf]

nature portfolio

Corresponding author(s): Minming Zhang

Last updated by author(s): Sep 26, 2022

Reporting Summary

Nature Portfolio wishes to improve the reproducibility of the work that we publish. This form provides structure for consistency and transparency in reporting. For further information on Nature Portfolio policies, see our Editorial Policies and the Editorial Policy Checklist.

Statistics

For all statistical analyses, confirm that the following items are present in the figure legend, table legend, main text, or Methods section.

n/a	Confirmed	
☐	☒	The exact sample size (n) for each experimental group/condition, given as a discrete number and unit of measurement
☐	☒	A statement on whether measurements were taken from distinct samples or whether the same sample was measured repeatedly
☐	☒	The statistical test(s) used AND whether they are one- or two-sided<br>*Only common tests should be described solely by name; describe more complex techniques in the Methods section.*
☐	☒	A description of all covariates tested
☐	☒	A description of any assumptions or corrections, such as tests of normality and adjustment for multiple comparisons
☐	☒	A full description of the statistical parameters including central tendency (e.g. means) or other basic estimates (e.g. regression coefficient) AND variation (e.g. standard deviation) or associated estimates of uncertainty (e.g. confidence intervals)
☐	☒	For null hypothesis testing, the test statistic (e.g. F, t, r) with confidence intervals, effect sizes, degrees of freedom and P value noted<br>*Give P values as exact values whenever suitable.*
☒	☐	For Bayesian analysis, information on the choice of priors and Markov chain Monte Carlo settings
☒	☐	For hierarchical and complex designs, identification of the appropriate level for tests and full reporting of outcomes
☐	☒	Estimates of effect sizes (e.g. Cohen's d, Pearson's r), indicating how they were calculated

Our web collection on statistics for biologists contains articles on many of the points above.

Software and code

Policy information about availability of computer code

Data collection	Diffusion images were preprocessed using FMRIB Software Library (FSL, https://fsl.fmrib.ox.ac.uk/fsl/fslwiki/), including the brain extraction and the correction of eddy current distortion and inter-volume head motion. the resulting diffusion data were fitted to the NODDI model using the NODDI MatLab Toolbox (http://www.nitrc.org/projects/noddi_toolbox) to generate NDI maps, ODI maps, and fiso maps.Cortical reconstruction and estimation of cortical thickness were performed using the FreeSurfer software (version 6.0.0, http://surfer.nmr.mgh.harvard.edu/fswiki).
Data analysis	GBSS voxel-wise analyses on the skeletonized NODDI parameter maps were performed with FSL's randomize by using nonparametric permutation analyses (n = 5000 permutations).Cortical thickness analyses were assessed vertex-wise using a GLM and the level of statistical significance was evaluated using a cluster-wise P (CWP) value correction procedure for multiple comparisons with cluster-based Monte–Carlo simulation using 5000 permutations.

For manuscripts utilizing custom algorithms or software that are central to the research but not yet described in published literature, software must be made available to editors and reviewers. We strongly encourage code deposition in a community repository (e.g. GitHub). See the Nature Portfolio guidelines for submitting code & software for further information.

Data

Policy information about availability of data

All manuscripts must include a data availability statement. This statement should provide the following information, where applicable:

- Accession codes, unique identifiers, or web links for publicly available datasets
- A description of any restrictions on data availability
- For clinical datasets or third party data, please ensure that the statement adheres to our policy

The data used in this manuscript are available from the corresponding author upon reasonable request.

Field-specific reporting

Please select the one below that is the best fit for your research. If you are not sure, read the appropriate sections before making your selection.

☒ Life sciences ☐ Behavioural & social sciences ☐ Ecological, evolutionary & environmental sciences

For a reference copy of the document with all sections, see nature.com/documents/nr-reporting-summary-flat.pdf

Life sciences study design

All studies must disclose on these points even when the disclosure is negative.

Sample size	76 PD patients and 32 HCs
Data exclusions	Subjects with a history of other neurologic or psychiatric disorders, brain trauma, general exclusion criteria for MR scanning, or PD patients meeting the diagnostic criteria for dementia were excluded from this study.
Replication	Cortical microstructural alterations in PD-NC and PD-MCI, relative to HC, the patterns with cluster-based thresholding method were similar with those adjusted by using with FWE multiple-comparison correction
Randomization	No randomization was performed. This study is an observational study without any intervention.
Blinding	Yes

Reporting for specific materials, systems and methods

We require information from authors about some types of materials, experimental systems and methods used in many studies. Here, indicate whether each material, system or method listed is relevant to your study. If you are not sure if a list item applies to your research, read the appropriate section before selecting a response.

Materials & experimental systems

n/a	Involved in the study	
☒	☐	Antibodies
☒	☐	Eukaryotic cell lines
☒	☐	Palaeontology and archaeology
☒	☐	Animals and other organisms
☐	☒	Human research participants
☒	☐	Clinical data
☒	☐	Dual use research of concern

Methods

n/a	Involved in the study	
☒	☐	ChIP-seq
☒	☐	Flow cytometry
☐	☒	MRI-based neuroimaging

Human research participants

Policy information about studies involving human research participants

Population characteristics	Clinical variables of HC (n=32), PD-NC (n=38) ,PD-MCI (n=38) are listed below<br>Age, years 59.11±7.13, 55.75±9.23, 59.69±7.80<br>Sex (F/M) 19/13, 23/15, 28/10
Recruitment	All the PD patients were diagnosed according to the criteria of UK Parkinson Disease Society Brain Bank by a senior neurologist
Ethics oversight	The data used in this manuscript are available from the corresponding author upon reasonable request.

Note that full information on the approval of the study protocol must also be provided in the manuscript.

Magnetic resonance imaging

Experimental design

Design type	Resting state
Design specifications	All participants were scanned in resting state by using a 3.0-Tesla MRI scanner (GE Discovery 750) equipped with an 8-channel head coil at the Department of Radiology of the Second Affiliated Hospital of the Zhejiang University School of Medicine. During MRI scanning, the head was stabilized using restraining foam pads, and earplugs were provided to reduce the noise.
Behavioral performance measures	There is no behavioral performance measure of participant.

Acquisition

Imaging type(s)	Two-shell diffusion images and 3D T1 images
Field strength	3.0 Tesla
Sequence & imaging parameters	Two-shell diffusion images were acquired using spin-echo echo-planar imaging sequence with 30 gradient directions for each non-zero b value (b value = 1000 s/mm2, 30 directions; b value = 2000 s/mm2, 30 directions); The sequence parameters were as follows: repetition time (TR) = 5000 ms; echo time (TE) = 94 ms; flip angle = 90°; field of view (FOV) = 256 × 256 mm2; matrix = 128 × 128; slice thickness = 4 mm; slice gap = 0 mm; 34 interleaved axial slices and acquisition time was 5 minutes 20 seconds. Three dimensional T1-weighted images were acquired using fast spoiled gradient recalled sequence: TR = 7.3 ms; TE = 3.0 ms; inversion time (TI) = 450 ms; flip angle = 11; FOV = 260 × 260 mm2; matrix = 256 × 256; slice thickness = 1.2 mm; slice gap = 0 mm; 196 continuous sagittal slices and acquisition time was 5 minutes 53 seconds.
Area of acquisition	Whole brain

Diffusion MRI ☒ Used ☐ Not used

Parameters: Two-shell diffusion images were acquired using spin-echo echo-planar imaging sequence with 30 gradient directions for each non-zero b value (b value = 1000 s/mm2, 30 directions; b value = 2000 s/mm2, 30 directions); The sequence parameters were as follows: repetition time (TR) = 5000 ms; echo time (TE) = 94 ms; flip angle = 90°; field of view (FOV) = 256 × 256 mm2; matrix = 128 × 128; slice thickness = 4 mm; slice gap = 0 mm; 34 interleaved axial slices and acquisition time was 5 minutes 20 seconds.

Preprocessing

Preprocessing software	FMRIB Software Library (FSL, http://www.fmrib.ox.ac.uk/fsl/) and FreeSurfer software (version 6.0.0, http://surfer.nmr.mgh.harvard.edu/fswiki)
Normalization	each participant “pseudo T1-weighted” images were registered to OASIS-30_Atropos_template with ANTs.
Normalization template	OASIS-30_Atropos_template
Noise and artifact removal	Eddy-current-induced distortion and head-motion artifacts correction using FSL’s “eddy_correct” tool
Volume censoring	No volume censorship has been done

Statistical modeling & inference

Model type and settings	GBSS voxel-wise analyses on the skeletonized NODDI parameter maps were performed with FSL’s randomize;Cortical thickness analyses were assessed vertex-wise using a GLM and the level of statistical significance was evaluated using a cluster-wise P (CWP) value correction procedure for multiple comparisons with cluster-based Monte–Carlo simulation using 5000 permutations.
Effect(s) tested	No effect test has been done

Specify type of analysis: ☒ Whole brain ☐ ROI-based ☐ Both

Statistic type for inference (See Eklund et al. 2016)	voxel-wise with GBSS
Correction	family-wise error (FWE) method and cluster-based thresholding method

Models & analysis

n/a	Involved in the study
☒	☐ Functional and/or effective connectivity
☒	☐ Graph analysis
☒	☐ Multivariate modeling or predictive analysis
